# Gender differences and outcome of melanoma patients

**DOI:** 10.1186/1479-5876-13-S1-P13

**Published:** 2015-01-15

**Authors:** Francesca Morgese, Rossana Berardi, Caterina Sampaolesi, Mariangela Torniai, Giulia Marcantognini, Alfredo Giacchetti, Stefano Serresi, Azzurra Onofri, Alessandro Bittoni, Alberta Pilone, Giuseppe Ricotti, Stefano Cascinu

**Affiliations:** 1 Clinica di Oncologia Medica-Università Politecnica delle Marche-Azienda Ospedaliero-Universitaria Umberto I Ancona, Italy; 2 U.O. Dermatologia INRCA/IRCCS- Ancona, Italy

## Background

In the United States, 80,000 cases of melanoma are expected in 2014, representing 5% and 4% of all diagnosis of cancer in men and women, respectively.

In Italy in 2013, there were an estimated 10,500 cases of cutaneous melanoma, with an incidence of 14.3 cases per 100,000 male population and 13.6 cases per 100,000 female population.

Female melanoma patients generally exhibit significantly longer survival than male patients.

The aim of this study is to evaluate the role of gender in survival of melanoma patients and the relationship between gender and pathologic features of the neoplasm.

## Materials and methods

In this retrospective study, we examined 1,023 consecutive patients treated at the Department of Medical Oncology and at the Department INRCA-IRCCS of Dermatology in Ancona, Italy from February 1987 to March 2014. Survival analysis was conducted via Kaplan-Meier product-limit method and the Mantel-Haenszel log-rank test was employed to compare survival among groups. A significance level was set at a 0.05 value and all *p* values were two-sided.

## Results

Of the total of 1,023 patients examined, 47.6% were men and 52.4% were women.

Pathological features discerned by gender were the following. Thin Breslow thickness (pT1) was more common in women versus men (45% vs 38.6%); Breslow thickness >1 mm (pT2) represented 16% of melanomas both in women and men; but thick melanomas (pT3-pT4) resulted more frequent in men. The presence of histologic ulceration in melanoma was approximately 80% and 9.7% in men and women melanoma respectively. Finally “non brisk” TILs pattern was founded comparable in men and women (25%), rather absent TILs pattern proved as a result more characteristic of men melanoma.

Subgroups analysis showed that women had a significant advantage in 12-year disease free survival (DFS) and 12-year overall survival (OS) adjusted for Breslow thickness (T1-T2 melanomas, p<0.001), presence of histologic ulceration (p<0.001), absent regression (p<0.001), “non brisk” TILs pattern (DFS and OS, respectively p=0.005 and p=0.009) and “absent” TILs melanomas (p<0.001).

Globally, a significant female advantage was observed for overall survival (p<0.001).

## Conclusions

Our results show that women have a consistent and independent survival advantage compared with men after adjusting for many variables indicating that factors other than pathologic features reduce mortality risk in female melanoma patients. We aim to further investigation about possible biologic sex differences in tumor-host interactions.

